# A governance framework for facilitating cross-agency data sharing

**DOI:** 10.23889/ijpds.v9i5.2564

**Published:** 2024-09-18

**Authors:** Katherine O'Sullivan, Joanne Lumsden, Caroline Anderson, Corri Black, William Ball, Katie Wilde

**Affiliations:** 1 University of Aberdeen; 2 Aberdeen City Council; 3 NHS Grampian; 4 Robert Gordon University

Linking data across local authorities and health boards has the potential to reduce inequalities and improve outcomes. However, cross-agency data sharing leads to complexities around data ownership, legal conditions for processing, organisational controls and risk assessments to ensure privacy and confidentiality of subjects whilst facilitating research and service improvements. We share a governance framework for cross-agency data sharing between a local authority and regional health board in Scotland.

A cross-sector Child Neglect working group was established with representation from public sector agencies, the NHS, University researchers and a Trusted Research Environment to support research on children’s mental health prescribing and secondary specialist services provision and linking to the Aberdeen City Council Child Protection Register. Lessons learned were captured throughout the governance, data transfer and data linkage phases.

Communication is the most important driver, with key individuals, deputies and routes of escalation agreed and involved throughout for clarity of roles and expectation setting. Key lessons learned include requiring input from all parties at the project initiation stage, particularly IG teams, including agreements on necessary templates. Knowledge exchange is required between the research team, data owners and data processors to ensure research protocol requirements are correctly understood and data transfer is supported. If possible, internal and/or external validation of data should also be performed before data is linked.

Cross-agency data sharing is a vital means to understanding and improving outcomes for individuals and populations. Critical to this is establishing an action plan and formalised project process to achieve outcomes.

